# Scoping review on trauma and recovery in youth after natural disasters: what Europe can learn from natural disasters around the world

**DOI:** 10.1007/s00787-022-01983-y

**Published:** 2022-04-15

**Authors:** Andreas Witt, Cedric Sachser, Jörg M. Fegert

**Affiliations:** Department of Child and Adolescent Psychiatry and Psychotherapy, University of Ulm, Steinhövelstraße 1, 89073 Ulm, Germany

**Keywords:** Natural disasters, Trauma, Recovery, Resilience, Children, Stepped care, Trajectories

## Abstract

In the last decade, Europe has seen a rise in natural disasters. Due to climate change, an increase of such events is predicted for the future. While natural disasters have been a rare phenomenon in Europe so far, other regions of the world, such as Central and North America or Southeast Asia, have regularly been affected by Hurricanes and Tsunamis. The aim of the current study is to synthesize the literature on child development in immediate stress, prolonged reactions, trauma, and recovery after natural disasters with a special focus on trajectories of (mal-)adaptation. In a literature search using PubMed, Psychinfo and EBSCOhost, 15 studies reporting about 11 independent samples, including 11,519 participants aged 3–18 years, were identified. All studies identified resilience, recovery, and chronic trajectories. There was also evidence for delayed or relapsing trajectories. The proportions of participants within each trajectory varied across studies, but the more favorable trajectories such as resilient or recovering trajectory were the most prevalent. The results suggested a more dynamic development within the first 12 months post-disaster. Female gender, a higher trauma exposure, more life events, less social support, and negative coping emerged as risk factors. Based on the results, a stepped care approach seems useful for the treatment of victims of natural disasters. This may support victims in their recovery and strengthen their resilience. As mental health responses to disasters vary, a coordinated screening process is necessary, to plan interventions and to detect delayed or chronic trauma responses and initiate effective interventions.

## Background

Lately, Europe has seen a number of natural disasters. Only in 2021, Europe has faced large floods in Belgium and Germany, and wildfires, especially devastating in Greece, Turkey, and throughout southern European countries. Natural disasters are defined as major adverse events resulting from natural processes of the earth [1]. They have the capacity to negatively impact large groups of individuals at once, often causing destruction and injuries, as well as mortality [2, 3].

While Europe has been less affected, but only come to face such events in the recent years, other regions of the world have been affected more intensely. E.g., the U.S. faces a Hurricane and Tornado season each year and the country has been affected by devastating natural disasters such as Hurricane Katrina in 2005. But also, other regions of the world have been affected. To name one was the Indian Ocean Tsunami that hit the coasts of India, Indonesia, Maldives, Sri Lanka and Thailand, Malaysia, Myanmar, Seychelles, Somalia, and the United Republic of Tanzania on Christmas 2004. The consequences were 186,983 people killed. Hundreds of thousands of persons were displaced and over three million persons were affected, half of whom lost their sources of livelihood [4].

Due to climate change, environmental hazards are set to increase in Europe [5]. Therefore, for example, the area simultaneously affected in EU has grown by 50% in the past 50 years [6], leading to an estimated fivefold increase in costs for flooding by 2050 [7]. Research from 2020 indicates that recent floodings have been exceptional and may be related to climate change [8]. This research has analyzed almost 10,000 river floods in Europe in the past 500 years and has shown that recent patterns of flooding are exceptional in extent. Previous flood-rich periods have often been linked to cooler average temperatures. Today’s flooding takes place in the context of warmer air and ocean temperatures. Also, with global warming, an increase in slow-moving storms which have the potential for high rainfall accumulations can be expected. This is very relevant to the recent flooding seen in Germany and Belgium, which highlights the devastating impacts of slow-moving storms. Research suggests that slow-moving rainstorms could be 14 times more frequent by the end of the century [9]. This study suggests that changes to extreme storms will be significant and cause an increase in the frequency of devastating flooding across Europe.

While some areas experience vast amounts of rain in short periods of time, other areas experience water shortages and droughts. Research indicates that climate change will substantially increase the severity and length of droughts in Europe by the end of the century [10]. And in turn, droughts further exacerbate the risk of wildfires [11].

Clemens and colleagues [12] have reviewed the mental health sequalae of climate change. They conclude that climate may affect children and adolescent in three ways: (1) Direct consequences, such as natural disasters; (2) Indirect consequences, such as loss of land, flight and migration, exposure to violence, change of social, ecological, economic, or cultural environment; and (3) The increasing awareness of the existential dimension of climate change can influence their mental health. Their results indicate that climate change represents a serious threat to mental health. They argue that children´s rights, mental health, and climate change should not be seen as separate aspects, but need to be brought together under one perspective to address the major challenges in the future of children and adolescents.

Youth are considered to be most affected by disasters (e.g., hurricanes, floods, wildfires, and droughts) around the world [13–15]. It is estimated that worldwide, 175 million children are exposed to disasters including floods, cyclones, droughts, and earthquakes each year [15]. Youth exposed to disasters are at risk for developing posttraumatic stress symptoms (PTSS) with greatly varying prevalence rates between 1 and 95% in children and adolescent survivors of natural disasters [16]. Longitudinal studies show rather persistent psychopathology over time and higher risk for psychiatric impairment in adulthood. However, not all who report initially elevated posttraumatic stress symptoms report persistent levels that last beyond the first three to six months after the event. It is therefore crucial to understand how youth develop after such events and why they differ in their adaptation patterns.

A growing body of literature documents heterogeneity among adults’ responses to potentially traumatic events [17, 18]. Across studies, these trajectories are chronic, recovery, resilience, and delayed. In this context, the trajectories are described by the pattern they exhibit over the observational period. A resilient trajectory is usually defined as a stable trajectory of healthy functioning after a highly adverse event [19]. In contrast, a chronic trajectory is characterized by a stable trajectory of psychopathology, usually higher posttraumatic stress symptoms, or maladaptation after adverse events. Recovery connotes trajectories in which normal functioning temporarily gives way to threshold or sub-threshold psychopathology, usually for longer periods of at least several months, and then returning to pre-event levels [19]. A delayed trajectory is marked by the initial sub-threshold psychopathology or healthy functioning, followed by an increase in psychopathology, mostly measured by an increase of posttraumatic stress symptoms at later time points during the observational period [13].

Less is known about the adaptation processes of children and adolescents after natural disaster. In their literature review, Lai and colleagues [13] examined trajectories of posttraumatic stress symptoms and predictors among children after natural disasters. Their results indicate, that mostly three trajectories (resilient, recovery, and chronic) were identified. The resilient trajectory was the most prevalent. Female gender, disaster exposure, negative coping, and lack of social support were significant risk factors for chronic trajectories. However, different outcomes were not examined. Therefore, the aim of the present manuscript is to synthesize the literature on the psychological consequences and recovery after natural disasters and to update the review of Lai and colleagues [13] and to expand the review to outcomes other than posttraumatic stress symptoms. Factors associated with more favorable developments, such as resilient or recovery trajectories are examined. Based on the results implications for interventions after natural disasters are discussed.

## Method

A literature search was conducted to examine trajectories of development in children following disasters. The search was broad, as not only trajectories of symptoms of posttraumatic stress disorders were included but also other indicators of psychopathology and adaptation after natural disasters (e.g., hurricanes, floods). Articles up to August, 2021 were included in the search. No start date was applied to allow inclusion of older studies. The literature search was conducted using PubMED, PsychInfo, and all databases available through the EBSCOhost with Boolean operators. The following terms and synonyms were used: natural disasters and child* and adolescen* in combination with trajectory, trauma, recovery, resilience, and psychological consequences. Filters were used in each database to search for manuscripts limited to participants up to the age of 18 years. In PubMED, the search included medical subject headings with Boolean operators using the terms noted earlier. To ensure search inclusivity, articles that cited studies found in our search were considered as well. Reference lists of identified articles were hand searched for additional relevant studies.

The literature search was conducted by the first author of the study. The titles and abstracts of each record were screened for eligibility by the first author. The full-text articles were independently reviewed by two researchers to identify records meeting our eligibility criteria. The extraction of data for the descriptive analyses was conducted by the first author. Quality of studies was not assessed systematically and was therefore not included in analyses.

**Fig. 1:**
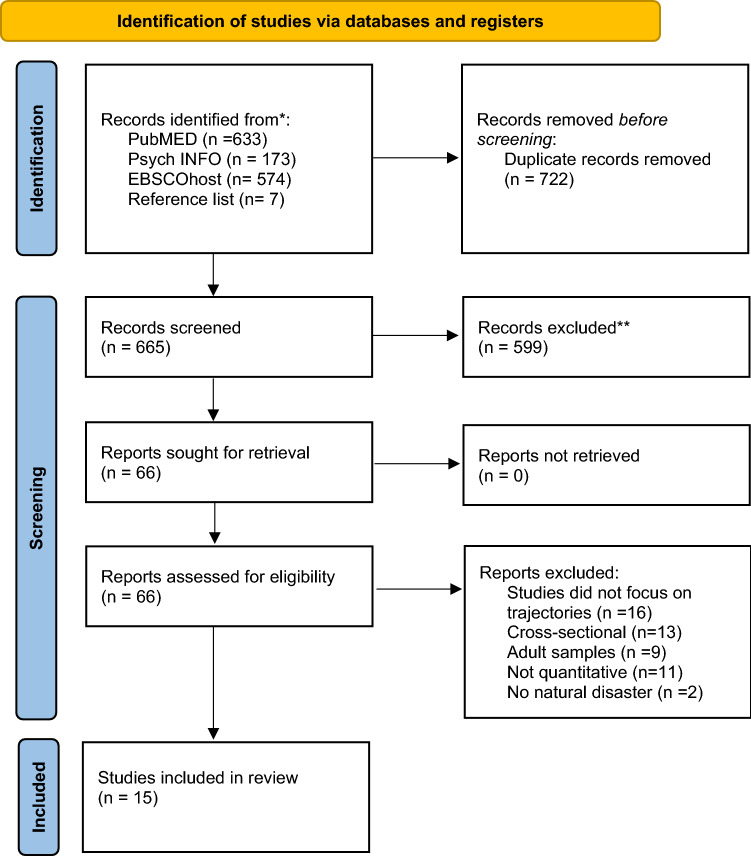
PRISMA flow diagram of identification and inclusion of records

Studies were included if they (1) were longitudinal, (2) examined trajectories of (mal-)adaption, (3) focused on development after natural disasters, (4) included participants up to the age of 18 years of age, (5) were written in English or German, (6) were quantitative studies, and (7) were published in a peer-reviewed journal. The identification process of studies included in the present review according to the PRISMA statement [20] is presented in Fig. 1.

After an initial screening of the titles of all records, the abstracts and partly the methods section of each identified article were read to determine whether the record met the inclusion criteria, resulting in 15 articles for inclusion in this review. The type of natural disaster, age of the study population, time points of assessment, symptoms assessed, and number of trajectories and percent of the sample following each trajectory were noted. The primary goal was to evaluate trajectory patterns of (mal-) adaptation after natural disasters among children and adolescents.

## Results

This review identified 15 empirical studies on youth trajectories of (mal-)adaptation following natural disasters. In total, the studies included 11,519 (*n* = 141 to *n* = 4619) children aged 3–18 years. The characteristics of all identified and included studies are presented in Table 1. The largest proportion of studies used latent growth mixture modeling (GMM) to determine the number of trajectories following natural disasters. Only one study [21] used latent transitioning analysis (LTA) to determine the development of children and adolescents after natural disasters. The 15 identified studies reported results from 13 different populations. Three articles [22–24] examined different outcomes of the same sample of 1573 children that experienced the Wenchuan Earthquake in China, and two articles [25, 26] examined different outcomes of the same sample of 391 children and adolescents who also experienced the Wenchuan Earthquake in China.

**Table 1:** Summary of studies included in the literature review

Study	Disaster	Baseline ages (years)	Sample	Number of participants	Assessments (months post-disaster)	Measure of PTSS	Additional measure(s)	Number and label of trajectories
Chen and Wu [21]	Ya’an Earthquake, China	*M* = 12.71 (SD = 2.78)	Convenience school sample from grades 4,5,7,8,10, 11 No information on response rate	*N* = 757	8 and 20 months after the earthquake	Child PTSD Symptom Scale (CPSS)	Post-traumatic Growth Inventory (PTG)	3: 8 months: resilient (21.1%), thriving (56.9%), and struggling (21.9%) 20 months: resilient (12.0%) thriving (72.0%), struggling (16.0%)
Fan et al. [22]	Wenchuan Earthquake, China	12–16 years (7th graders *n* = 216, initial *M* = 12.30, SD = 0.53 years; 10th graders *n* = 1357, initial *M* = 15.44, SD = 0.67 years)	Convenience School sample from two schools grade 7 and 10 No information on response rate	*N* = 1573	6, 12, 18 and 24 months after earthquake	Post-traumatic Stress Disorder Self-Rating Scale (PTSD-SS)	The Adolescent Self-Rating Life Events Checklist (ASLEC) The Social Support Rate Scale (SSRS) The Simplified Coping Style Questionnaire (SCSQ)	5: resistance (resilient, 65.3%), recovery (20.0%), relapsing/remitting (3.3%), delayed dysfunction (4.2%) and chronic dysfunction (7.2%)
Shi et al. [23]	Wenchuan Earthquake, China	12–16 years (*M* = 15.00)	Convenience School sample from two schools grade 7 and 10 No information on response rate	*N* = 1573	6, 12, 18, and 24 months after earthquake	–	Screen for Child Anxiety Related Emotional Disorders (SCARED) Adolescent Self-Rating Life Events Checklist (ASRLEC) Social Support Rating Scale (SSRS) Resilience Scale (RS)	4: resistance (resilient, 65%), recovery (9.1%), delayed-onset anxiety (3.2%), and chronic anxiety (22.7%)
Qin et al. [24]	Wenchuan Earthquake, China	12–16 years (*M* = 15.00)	Convenience School sample from two schools grade 7 and 10 No information on response rate	*N* = 1573	6, 18, and 30 months after earthquake	PTSD Self-Rating Scale (PTSD-SS)	Strengths and Difficulties Questionnaire (SDQ) Screen for Child Anxiety Related Emotional Disorders (SCARED) Depression Self-Rating Scale for Children (DSRSC) Adolescent Self-Rating Life Events Checklist (ASLEC) Social Support Rate Scale (SSRS) Coping Style Questionnaire (SCSQ)	Procsocial Behavior Trajectories 4: high/enhancing (35.0%), (b) high/stable (29.4%), (c) low/declining (33.6%), and (d) low/steeply declining (2.0%)
Zhou et al. [25, 26]	Wenchuan Earthquake, China	12–19 years (*M* = 15.28, SD = 1.81)	Convenience School sample from four middle schools According to the authors all students in the selected classes participated	*N* = 391	12, 18, 24, and 30 months after earthquake	Child PTSD Symptom Scale (CPSS)	Academic Burnout Inventory (ABI)	3: moderate-stable (resilient, 81.6%), decreasing (recovery, 8.7%), and increasing trajectories (delayed, 9.7%) Academic burnout Trajectories: 3: Increasing (3.9%), low (resilient, 85.4%), and decreasing (recovery, 10.7%)
Liang et al. [27]	Wenchuan Earthquake, China	*M* = 12.5 years (SD = 1.17)	Convenience School sample from grades 4 and 6 No information on response rate	*N* = 300	4, 16, 29, 40, and 52 months after the earthquake	University of California, Los Angeles, Post-traumatic Stress Disorder Reaction Index (UCLA PTSD-RI)	Child Depression Inventory (CDI	3: resilient (74.9%), recovery (7.5%) and relapsing (17.7%) Depression: 2: resilient (66.2%) and chronic (33.8%)
Cheng et al., [28]	Lushan Earthquake, China	9–17 years (*M* = 12.5, SD = 1.69)	Convenience School sample from grades 4, 5, 6, 7, 8, and 9 No information on response rate	*N* = 304	1.5, 6, 12, 24 and 48 months after the earthquake	The University of California at Los Angeles Posttraumatic Stress Disorder Reaction Index for DSM-IV (UCLA PTSD-RI)	The Acute Stress Disorder Scale (ASDS)	4: resilience (53.8%), low symptoms (32.6%), recovery (7.0%), and chronic dysfunction (6.6%)
Kronenberg et al. [29]	Hurricane Katrina, USA	9–18 years (*M* = 14.27 years, SD = 2.13)	Convenience School sample from grades 4, 5, 6, 7, 8, 9, 10, 11, and 12 Response rate of 67%	*n* = 387	24, 36 months after the hurricane	NCTSN Hurricane Assessment and Referral Tool for Children and Adolescents (NCTSN)	Depression with NCTSN Hurricane Assessment and Referral Tool for Children and Adolescents (NCTSN)	5: stress resistant (resilient, 45.2%), Recovery (27.1%), delayed breakdown (4.7%), and breakdown without recovery (chronic, 23.0%)
Self-Brown et al., [30]	Hurricane Katrina, USA	8–16 years (*M* = 11 years)	Convenience School sample from 6 schools Response rate of 35%	*N* = 426	13, 19 and 25 months after hurricane	University of California, Los Angeles, Posttraumatic Stress Disorder Reaction Index (UCLA-PTSD-RI)	Hurricane-related traumatic events scale screen for adolescent/child violence exposure (SAVE, KID-SAVE) social support scale for children (SSSC)	3: Chronic (4%), recovering (27%), and (c) resilient (70%)
La Greca et al., [31]	Hurricane Andrew, USA	8–11 years (*M* = 9.33, SD = 0.98)	Convenience School sample from 3 schools from grades 3, 4 and 5 No information on response rate	*N* = 568	3, 7, and 10 months after Hurricane	The PTSD-RI Hurricane-Related Traumatic Experiences (HURTE)	Anxiety: The Revised Children’s Manifest Anxiety Scale (RCMAS) Social support: Social Support Scale for Children (SSSC) Coping: Kidcope Life Events: Life Events Schedule	3: resilient (37%), recovering (43%), and chronic distress (20%)
Lai et al. [33]	Hurricanes Andrew [1992], Charley [2004], Ike [2005], and Katrina [2008], USA	6–16 years (*M* = 9.61, SD = 1.60)	Pooled data of convenience samples from elementary, middle and high schools No information on response rates	*N* = 1707	3 to 26 months after the disasters	University of California, Los Angeles, Posttraumatic Stress Disorder Reaction Index (UCLA PTSD-RI)		4: chronic (10%), recovery (23%), moderate-stable (33%), and low-decreasing (recovery, 34%)
Osofsky et al. [34]	Hurricane Katrina, Hurricane Gustav, Oil Spill, USA	3–18 years	Convenience School sample from pre-kindergarten to grade 12 as part of an ongoing community school-based screening No information on response rate	*N* = 4619	12, 24, 37, 49 months after hurricane	LSU KIDS (Louisiana State University Health Sciences Center Katrina Inspired Disaster Screenings) a modified version of the NCTSN Disaster Assessment and Referral Tool for Children and Adolescents (NCTSN)	Self developed measures for hurricane exposure and oil spill stress	4: stable-low symptoms (resilience, 52%), steep declines following initial symptoms (recovery, 21%), increasing symptoms (delayed, 18%), stable-high symptoms (chronic, 9%)
Weems, et al., 2014 [35]	Hurricanes Katrina and Gustav, USA	8–15 years	Convenience school sample from 1 school from grade through grade 12 No information on response rate	*N* = 141	24 months post-Katrina/12 months pre Gustav, 30 months post Katrina/ 6 months pre Gustav, 36 months post Katrina/ 1 month post-Gustav	Post-Traumatic Stress Reaction Index for Children (PTSD-RI)	Children’s Coping Strategies Checklist	5: stable low (41%), moderate (23%), increasers (delayed) (9%), stable high (chronic) (15%), decreasers (recovery) (10%)
McDonald et al., 2019 [32]	2011 Tuscaloosa Alabama Tornado, USA	9–13 years (*M* = 11.33 years)	Systematic school sample from 20 elementary schools; Two-gate screening procedure; No information on response rate	*n* = 346	3, 48 months after tornado	PTSD- Reaction Index (PTSD-RI) Tornado-Related Traumatic Experiences questionnaire (TORTE)	Alabama Parenting Questionnaire (APQ)	3: (1) recovery (15.9%); (2) stable and low (resilient) (76.9%); (3) stable and high (chronic) (7.2%)

*M *= mean age; SD = standard deviation; *n* = number

Only studies examining populations from China and USA were identified. Most of the studies (*n* = 12) examined trajectories following one event [21–32], but three studies examined trajectories after multiple events [33–35]. The main events examined were the Wenchuan Earthquake (*n* = 6), Hurricane Katrina (*n* = 5), Hurricane Andrew (*n* = 2), and Hurricane Gustav (*n* = 2). The studies had between two and five follow-up time points, and the follow-up period lay between one and 52 months after the disaster.

### Trajectories of posttraumatic stress symptoms

**Fig. 2:**
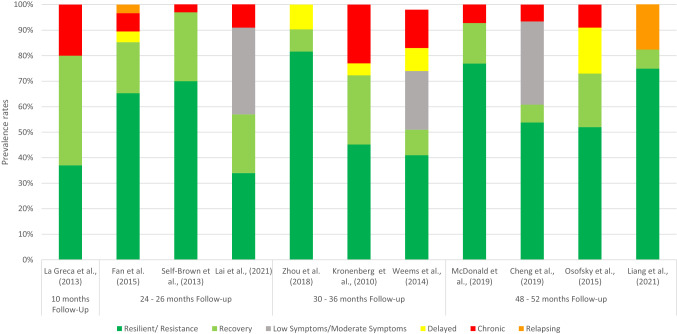
Posttraumatic stress symptom trajectories of children and adolescents after natural disasters identified in the literature

All studies that assessed posttraumatic stress symptoms identified resilience and recovery trajectories. Only one study [25] did not identify a chronic trajectory, which was common in all other studies. The results for a delayed trajectory identified in five studies [22, 25, 29, 34, 35] were mixed. Additionally, three studies identified a trajectory of low or moderate symptoms [28, 33, 35] and two studies identified a relapsing trajectory [22, 27]. The proportions of children falling into each trajectory varied widely across studies, but overall, resilience was the most prevalent trajectory (34%–81.6%). Taken together, the majority of participants either showed a resilient or recovering trajectory (51%–97%). In Fig. 2, the trajectories of posttraumatic stress symptoms identified in the literature are presented.

### Trajectories of (mal-)adaptation after natural disasters

**Fig. 3:**
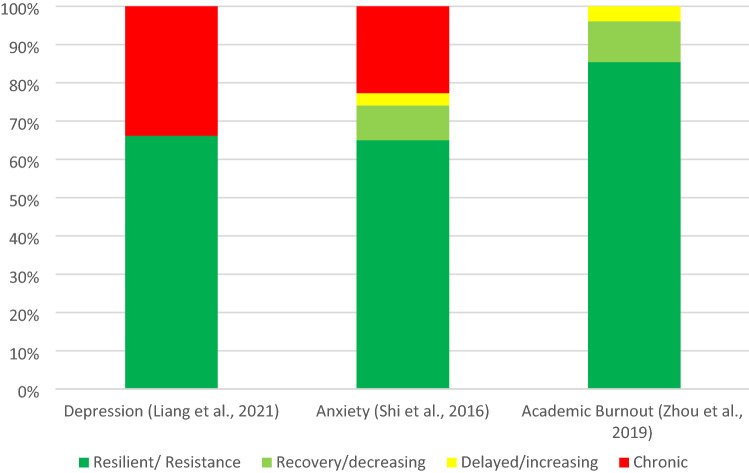
Mental health outcome trajectories of children and adolescents after natural disasters identified in the literature

Four studies focused on outcomes other than posttraumatic stress symptoms and examined trajectories of depression [27], anxiety [23], and academic burnout [26] after natural disasters. Only one study [24] focused on an adaptive outcome, such as prosocial behavior. For other mental health outcomes, such as depression or anxiety, a resilient trajectory was the most prevalent and evidence for other trajectories were mixed (see Fig. 3). The results from Qin and colleagues [24] focusing on prosocial behavior, indicate that a total of 74.4% of the sample show more adaptive trajectories with high enhancing and high stable trajectories of prosocial behavior.

### Short-term development

Four studies [22, 27, 28, 31] included time points within the first 16 months post-disaster allowing to examine more short-term developments. Fan and colleagues [22] identified an increase of symptoms between 6 and 12 months after the event, a decrease of symptoms in the recovery trajectory sets in after 12 months. Liang and colleagues [27] identified increases and decreases of symptoms within the first 16 months followed by a stable period. Then, after 40 months, a more dynamic development can be observed. Another study [28] identified increases and decreases in symptoms within the first 12 months post-disaster, again followed by a rather stable period of symptom trajectories. La Greca and colleagues [31] identified a decrease in symptoms within the first 12 months post-disaster.

### Long-term development

Four studies [27, 28, 32, 34] reported about a follow-up period that exceeded 48 months post-disaster. With 6.6%–9% of chronic trajectories, they reported relatively high number of participants that showed adaptation in the aftermath of the disasters. When looking at the results no clear pattern of when changes in adaptation occur can be identified. For example, the results of Cheng and colleagues [28] suggest that changes especially occur within the first 12 months. After that symptoms either decline in the chronic trajectory or remain stable within the other trajectories, while the results of Liang et al. [27] point in a similar direction, but suggest that changes may occur after 46 months post-disaster. On the other hand, the results of Osofsky et al., [34] and McDonald and colleagues [32] rather point to less dynamic trajectories with more static increases, decreases, or stable trajectories.

### Risk and protective factors

Except Lai and colleagues [33] all studies examined risk and protective factors. Therefore, predictors distinguishing between different trajectories, i.e., resilient vs. CHRONIC trajectories, were examined. The factors identified in the studies examining trajectories of posttraumatic stress symptoms are presented in Table 2. Across studies female gender, a higher trauma exposure (i.e., suffering injury, perceived life threat), a higher number of life events, less social support, and negative coping were associated with less-favorable outcome trajectories.

**Table 2:** Risk and protective factors identified by the studies included

Domain	Variable	Study	Comparison
Child characteristics	Female gender	Fan et al. [22]	Resistance (resilience) vs. non-resistance (all four other groups combined)
		Cheng et al. [28]	Resilience vs. chronic
		Kronenberg et al. [29]	Resilience vs. chronic
			Resilience vs. recovery
		La Greca et al. [31]	Resilience vs. chronic
			Resilience vs. recovery
		Chen and Wu [21]	Boys had a higher probability to transition from increasing to resilient class
	Having siblings	Fan et al. [22]	Chronic vs. recovery
	Younger age	Kronenberg et al. [29]	Resilience vs. chronic
			Resilience vs. delayed
			Resilience vs. recovery
		Chen and Wu [21]	Resilience vs. chronic
			Recovery vs. chronic
	General anxiety	La Greca et al. [31]	Resilience vs. chronic
			Resilience vs. recovery
			Recovery vs. chronic
Exposure variables	Suffered family members death/missing Witnessed traumatic scenes	Fan et al. [22]	Resistance (resilience) vs. non-resistance (all four other groups combined)
			Chronic vs. recovery
	Trauma exposure	Zhou et al. [25]	Resilience vs. recovery
			Delayed vs. recovery
		Liang et al. [27]	Resilience vs. relapsing
	Suffered injury	Cheng et al. [28]	Resilience vs. recovery
		Chen and Wu [21]	Resilience vs. chronic
			Recovery vs. chronic
	Immediate Loss/disruption	Self-Brown et al. [30]	Resilience vs. recovery
	Higher Hurricane exposure	Weems et al. [35]	Resilience vs. chronic
	Higher Hurricane and Oil Spill exposure	Osofsky et al. [34]	Resilience vs. chronic
			Recovery vs. chronic
	Perceived life threat, More life-threatening events, More loss-disruptions	McDonald et al. [32]	Resilience vs. recovery
	Experiencing fear	Chen and Wu [21]	Resilience vs. chronic
			Recovery vs. chronic
Pre trauma exposure	pre-disaster trauma exposure	McDonald et al. [32]	Resilience vs. chronic
	Previous trauma	Osofsky et al. [34]	Resilience vs. chronic
			Resilience vs. recovery
			Resilience vs. delayed
		Kronenberg et al. [29]	Resilience vs. recovery
			Recovery vs. chronic
			Resilience vs. chronic
		Liang et al. [27]	Resilience vs. recovery
Post disaster exposure	More negative life events	Fan et al. [22]	Resistance (resilience) vs. non-resistance (all four other groups combined)
			Relapse vs. recovery
			Delayed vs. recovery
			Chronic vs. recovery
	Post-hurricane major loss or trauma	Kronenberg et al. [29]	Resilience vs. chronic
			Recovery vs. chronic
			Resilience vs. recovery
Family, friend, financial, housing, neighborhood violence problems	Family, friend, financial, housing, neighborhood violence problems	Kronenberg et al. [29]	Resilience vs. chronic
			Recovery vs. chronic
			Resilience vs. recovery
	Community violence	Self-Brown et al. [30]	Resilience vs. Recovery
			Resilience vs. chronic
	Family income	McDonald et al. [32]	Resilience vs. chronic
Relationships/support	Social support	La Greca et al. [31]	Recovery vs. chronic
	Peer social support	Self-Brown et al. [30]	Recovery vs. chronic
			Resilience vs. chronic
	Family connectedness	Kronenberg et al. [29]	Recovery vs. chronic
	Bad relationship to father	Cheng et al. [28]	Resilience vs. chronic
		Fan et al. [22]	Chronic vs. recovery
			Relapse vs. recovery
	Consulting with health counselor or therapist	Kronenberg et al. [29]	Resilience vs. chronic
			Recovery vs. chronic
Coping	Less positive more negative coping	Fan et al. [22]	Resistance (resilience) vs. non-resistance (all four other groups combined)
	Blame/anger coping	Kronenberg et al. [29]	Recovery vs. chronic
			Resilience vs. chronic
			Recovery vs. chronic
	Avoidant coping	Weems et al. [35]	Resilience vs. delayed

The four studies that examined trajectories of outcomes other than posttraumatic stress symptoms [23, 24, 26, 27] also examined predictors of trajectories. Liang and colleagues [27] identified an older age, and poorer parental relationship to be associated with a chronic depression trajectory as compared to a resilient depression trajectory. For anxiety gender, injury of family members, negative life events, social support, and trait resilience were significant predictors of a resilient versus a chronic trajectory [23]. Zhou and colleagues [26] examined the role of specific posttraumatic stress symptom clusters on academic burnout. The results indicated that intrusive PTSD symptoms were more likely in the delayed trajectory, PTSD hyperarousal symptoms were more likely in the recovery and resilient trajectory, and avoidance PTSD symptoms were more likely in the recovery trajectory. Qin et al. [24] found that male gender increased the probability of belonging to the stable, slightly declining and sharply declining trajectories of prosocial behavior relative to the enhancing trajectory. Additionally, adolescents with a lower level of social support were more likely to fall in the stable and slightly decreasing trajectory rather than the enhancing trajectory.

## Discussion

The aim of the present study was to synthesize the literature on outcome trajectories in children and adolescents after natural disasters, and to identify risk and resilience factors for more favorable trajectories. Therefore, the results of 15 studies, based on 11 distinct samples and 11.519 participants between 3 and 18 years of age were analyzed. For the most part, the results of Lai and colleagues 2017 on posttraumatic stress symptoms trajectories after disasters were replicated with largely varying prevalence rates for the distinct trajectories. Across studies on trajectories of posttraumatic stress symptoms, a resilient, recovery, and chronic trajectory (except Zhou et al. [25]) were identified, with the resilient trajectory (37%–82%) being the most prevalent trajectory. Taken together, six of the eleven studies provided evidence for a delayed or relapsing trajectory. This indicates that a proportion of children (4%–18%) who experienced a natural disaster remain at risk underlining the need for a clinical follow-up.

The evidence for trajectories of other mental health outcomes, such as depression, was limited with only four studies examining outcomes other than posttraumatic stress symptoms. All studies identified a resilient trajectory, but generally, the results for different trajectories was mixed. While the results for depression trajectories indicated more stable developments [27], the results for anxiety and academic burnout trajectories also indicated more dynamic developments with participants recovering or showing delayed responses [23, 26]. Only one study examined trajectories of more adaptive outcomes [24]. The results on the trajectories of prosocial behavior indicate that the development of more adaptive outcomes, that may be part of a broader resilience concept, might look different than the usual resilience, recovery, delayed, and chronic trajectories that are expected for maladaptive outcomes [19], such as posttraumatic stress symptoms.

A second focus of the literature review was the identification of risk and protective factors associated with more favorable trajectories. The results of Lai and colleagues [15] were largely replicated. Female gender, a higher trauma exposure (i.e., suffering injury, perceived life threat), a higher number of life events, less social support, and negative coping were associated with less-favorable posttraumatic stress symptom trajectories. However, these results are not unexpected as these factors represent well-established risk and resilience factors for posttraumatic stress disorders [36] and the cumulation of negative life events increases the risk of maladaptation [37–39]. Especially, the assessment of peritraumatic factors, such as trauma load, the suffering of injuries, witnessing traumatic scenes, to have a close person being killed or missing, etc. can be easy to assess variables that may help to identify at risk populations and to support those in dealing with their experiences. Newly occurring traumatic events or the experience of negative life events may be especially linked to delayed or relapsing trajectories, as research underlines the impact of newly occurring negative events on the psychosocial development of children [40]. Therefore, the delayed response (i.e., the relapse trajectory) might rather represent a reaction to newly occurring and cumulation of events, rather than a delayed reaction to the initial event. This underlines the need for prevention measures of adverse life events.

Social support, and less negative coping, both being identified as protective factors [13, 41] could be relevant starting points for interventions for affected populations. Group interventions could be implemented in schools where populations have been affected by the disaster and teach about effective coping strategies and solicit peer social support. Additionally, risk communication and disaster education are considered important aspects of disaster preparedness [42]. The literature suggests that schools are a suitable place for risk communication, and that adolescents should be involved and engaged in the communication strategies [42]. Due to the high risk of persisting psychological impairment in survivors of disasters, psychosocial interventions for children and adolescents have been developed over the past years. In their meta-analysis and systematic review, Brown and colleagues [43] have analyzed psychological interventions for children and adolescents after man-made and natural disasters. They found that overall treatments showed high effect sizes in pre–post-comparisons (Hedges’ *g* = 1.34) and medium-effect sizes as compared with control conditions (Hedges’ *g* = 0.43). These treatments were trauma-focused cognitive–behavioral therapy (tf-CBT), eye movement desensitization and reprocessing (EMDR), narrative exposure therapy for children (KIDNET), and classroom-based interventions, which showed similar effect sizes. Pfefferbaum and colleagues [44] expanded the results of Brown and colleagues [43] by also reviewing the type of interventions (e.g., focused psychosocial support) and the settings (e.g., schools) or the context where the interventions were delivered. However, they identified an overall effect size of *g* = 0.57, indicating that interventions had a medium beneficial effect on posttraumatic stress symptoms and enhancing daily functioning. Therefore, highly effective interventions for children and adolescents who experienced natural disaster exist and should be made available for them.

As the results of the present review indicate a high heterogeneity in reactions to disasters, the question remains, how and when interventions should be delivered. The results indicate a dynamic development of posttraumatic stress symptoms within the first year, potentially within the first 6 months [28], followed by a more stable development afterward. However, longitudinal research that exceeds 48 months post-disaster (e.g., [27]), though scarce, indicates that after a longer period, dynamic development in terms of relapses is possible. Considering the available data assuming a dynamic development of symptoms within the first 12 months post-disaster a screening process with multiple assessments of posttraumatic stress symptoms within the first year seems useful. Assessments should be conducted right after the event, after 3, 6 and 12 months post-disaster to identify high-risk populations. Additionally, a stepped care approach seems useful to address the needs of children exposed to natural disasters. This stepped care approach should include a classroom-based intervention, which should be delivered within a short period after the event. This broad intervention could be delivered in classrooms to strengthen protective factors such as social support and should include psychoeducation about trauma and trauma reactions as well as negative and positive coping strategies to support resilience and recovery. However, schools might not always remain open after natural disasters; therefore, other modalities of delivering interventions should be considered. These may include online interventions and interventions in community places or shelters.

As the data indicate a dynamic development of posttraumatic stress symptoms within the first 6 months post-disaster the initial broad intervention should be followed by a phase of watchful waiting as suggested, for example by the NICE guidelines (National Institute for Health and Care Excellence (NICE), [45]). Results indicate that a large number of children and adolescents affected show adaptation in the aftermath of disaster. Those still exhibiting high levels of posttraumatic stress symptoms after a period of 6 months post-disaster should receive treatments that have been proven to be effective in this context, such as trauma-focused cognitive behavioral therapy [43].

### Limitations

First, this review included 15 studies. However, the studies included to examine mental health outcomes other than posttraumatic stress symptoms only comprised four studies with varying outcomes, limiting the generalizability of the findings. Clearly, more research on trajectories of mental health outcomes, other than posttraumatic stress symptoms are needed. Second, studies included in this review only focused on samples from USA and China. Even though it can be expected that results may be generalizable to different cultural contexts, more culturally diverse research is needed. Third, retention rates for individual studies and missing data handling were different for individual studies. It is possible that certain trajectories (e.g., delayed or chronic) may show different retention rates. Therefore, attrition rates for trajectories may influence our understanding of proportions of the different trajectory. Fourth, the studies included in this review used differing analytic strategies (e.g., Growth Mixture Modeling (GMM) and Latent Transitioning Analysis (LTA)) which may have also impacted our understanding of proportions of trajectories as described before. Additionally, an analysis on quality of the studies was not conducted. Fifth, risk and resilience factors have not been assessed purposefully in the individual studies and not uniformly. Therefore, more research on protective factors is needed to gain a more complete picture of factors influencing children’s development after natural disasters. Furthermore, a potential publication bias needs to be considered [46]. In the present review, only scientific literature that was published in peer-reviewed journals, as well as literature published in German or English was considered. Dissertations, other gray literature and literature in languages other than German or English were not included potentially leading to the fact that research on the topic might exist that is not being captured in the present manuscript. This might especially be the case in research including outcomes other than posttraumatic stress symptoms. However, the focus on peer-reviewed literature was due to the sophisticated statistical methods applied to assure the quality of the studies included in this review.

## Conclusion

The latest natural disasters Europe has faced, such as devastating wildfires in Greece and Turkey, as well as floods in Germany and Belgium are harbingers of what Europe will have to face over the next decades, as due to climate change, natural disasters are expected to increase in Europe. Youth are especially affected by such disasters. Research indicates a variety of responses toward trauma exposure including resilience, recovery, delayed, and chronic trajectories. More favorable responses, such as resilience and recovery trajectories are the most prevalent. However, a substantial proportion of children and adolescents experience chronic and delayed trauma responses. Most of the dynamic development of (mal-)adaptation seems to take place within the first 12 months post-disaster. A broad screening process especially for posttraumatic stress symptoms with different points assessments within the first year seems useful. Assessments should be conducted immediately after the event, after 3, 6, and 12 months post-disaster to identify high-risk populations. Additionally, a stepped care approach seems useful to address the needs of children exposed to natural disasters. This stepped care approach should include a classroom-based intervention shortly after the event including psychoeducation and teaching adaptive coping strategies to strengthen protective factors and adaptation processes. In places where schools do not remain open, other modalities of delivering an initial intervention, such as online resources, should be considered. This should be followed by a period of watchful waiting including active assessment of posttraumatic stress symptoms. Those still exhibiting high levels of posttraumatic stress symptoms after a period of 6 months post-disaster should receive effective treatments, such as trauma-focused cognitive behavioral therapy (tf-CBT, [47]).

## Data Availability

Data sharing is not applicable to this article as no new data were created or analyzed in this study.
